# Computational Discovery of Novel Chalcogenide Perovskites YbMX_3_ (M = Zr, Hf; X = S, Se) for Optoelectronics

**DOI:** 10.3390/molecules30071468

**Published:** 2025-03-26

**Authors:** Qingyu Li, Helong Wu, Weiguo Li, Jiming Zhang, Rongjian Sa

**Affiliations:** 1 College of Materials Science and Engineering, Fujian Normal University, Fuzhou 350007, China; lqy1207984388@163.com (Q.L.); li371541131@163.com (W.L.); 2 College of Materials and Chemical Engineering, Minjiang University, Fuzhou 350108, China; 13960095640@163.com; 3 Zhongpu Technology (Fuzhou) Co., Ltd., Fuzhou 350108, China

**Keywords:** novel chalcogenide perovskites, phase stability, mechanical behavior, optoelectronic properties, conversion efficiency

## Abstract

Chalcogenide perovskites have shown great potential for photovoltaic applications. Most researchers have begun to pay close attention to the crystal synthesis, phase stability, and optoelectronic properties of chalcogenide perovskites AMX_3_ (A = Ca, Sr, Ba; M = Ti, Zr, Hf, Sn; X = S, Se). At present, the A-site metal cations are mainly limited to alkaline earth metal cations in the literature. The replacement of the alkaline earth metal cations by Yb^2+^ is proposed as an alternative for chalcogenide perovskites. In this study, the phase stability, and mechanical, electronic, optical, and photovoltaic properties of novel chalcogenides YbMX_3_ (M = Zr, Hf; X = S, Se) are theoretically evaluated in detail for the first time. It is mentioned that YbZrS_3_ and YbHfS_3_ are marginally thermodynamically stable while YbZrSe_3_ and YbHfSe_3_ exhibit superior phase stability against decomposition. Good mechanical and dynamical stability of these chalcogenide perovskites are verified, and they are all ductile materials. The accurate electronic structure calculations suggest that the predicted direct bandgap of YbMSe_3_ (M = Zr, Hf) is within 1.3–1.7 eV. Additionally, the small effective mass and low exciton binding energy of YbMSe_3_ (M = Zr, Hf) are favorable for their photovoltaic applications. However, YbZrS_3_ and YbHfS_3_ show larger direct band gaps with a change from 1.92 to 2.27 eV. The optical and photovoltaic properties of these compounds are thoroughly studied. In accordance with their band gaps, YbZrSe_3_ and YbHfSe_3_ are discovered to exhibit high visible-light absorption coefficients. The maximum conversion efficiency analysis shows that YbMSe_3_ (M = Zr, Hf) can achieve an excellent efficiency, especially for YbZrSe_3_, whose efficiency can reach ~32% in a film thickness of 1 μm. Overall, our study uncovers that YbZrSe_3_ is an ideal stable photovoltaic material with a high efficiency comparable to those of lead-based halide perovskites.

## 1. Introduction

Organic-inorganic lead-based halide perovskites have attracted unprecedented attention over the past decade [1,2,3,4]. Since the initial study reported by Kojima et al. in 2009 [5], the power conversion efficiency (PCE) of this kind of material has shown a remarkable increase [6,7,8]. In 2023, the certified PCE of α-formamidinium lead iodide (FAPbI_3_ with FA = (NH_2_)_2_CH) has reached up to 25.73% for single-junction solar cells [9]. However, the large-scale commercial application of these materials is severely limited by two key factors: their inherent instability and the toxic lead element. The excellent optoelectronic properties and high stability of inorganic halide perovskites have been explored in recent years by employing theoretical and experimental methods [10,11,12,13,14,15,16]. The maximum conversion efficiency of CsPbI_3_-based solar cells was ~19% in 2019 [17], which is much lower than that of FAPbI_3_.

Extensive efforts have been made to identify alternative candidates with excellent properties and high efficiency. Recently, the synthesis, optoelectronic properties, and photovoltaic performance of a series of chalcogenide perovskites have been studied theoretically and experimentally [18,19,20,21,22,23,24,25,26,27,28,29,30,31,32,33,34,35]. The chalcogenide perovskites with the general formula AMX_3_ (A = Ca, Sr, Ba; M = Ti, Zr, Hf, Ce; X = S, Se, Te) can provide an ideal platform for material design because the property engineering is allowed by the substitution of the A-, M-, or X-site. For example, the distorted perovskites including CaTiS_3_, CaZrSe_3_, CaHfSe_3_, and BaZrS_3_ are identified as promising solar cell candidates owing to their suitable direct band gaps [33]. Perera et al. reported that BaZrS_3_ has an experimental optical band gap of 1.73 eV and excellent stability [34]. Based on predictions from theoretical calculations, the band gap of BaZrS_3_ can be tuned to an ideal value of 1.47 eV by the substitution of Zr with ~10% Ti, but it is difficult to synthesize this alloy as it is easily decomposed to the corresponding ternary secondary phases [32]. The optical properties and electronic transport of BaZrS_3_ are also examined on the basis of first-principle calculations [24]. The modifications of the optoelectronic properties of AZrS_3_ (A = Mg, Ca, Sr, Ba) have been studied by applying different hydrostatic pressures [21]. The improvements of the band gap and light absorption of AZrS_3_ (A = Ca, Sr, Ba) can be achieved by the substitution of S with Se [35]. CaZrSe_3_ is predicted to be a promising thermoelectric material in addition to its potential for photovoltaic performance [23]. Furthermore, lead-free inorganic ACeTe_3_ (A = Ca, Sr, Ba) is proposed as an excellent photovoltaic material because it has a suitable direct band gap, strong absorption coefficient, and high PCE [31]. Ju et al. further predicted that SrSnS_3_ and SrSnSe_3_ are desired candidates for photovoltaic devices with optimal direct band gaps (1.0–1.6 eV) and good absorption properties (~10^5^ cm^−1^) [36]. Recently, several groups have developed a solution-based synthesis route to obtain a high-quality thin film of BaZrS_3_ at lower temperature (~300−600 °C) [26,29], which is a crucial step forward for optoelectronic applications of chalcogenide perovskites. Through the comprehensive analysis of these results, chalcogenide perovskites have great potential for photovoltaics.

According to the investigation of theoretical and experimental reports, we find that the A-site metal cations are mainly limited to alkaline earth metal cations (such as Ca^2+^, Sr^2+^, and Ba^2+^). It is interesting that the structural, electronic, optical, and thermoelectric properties of perovskite-type AYbX_3_ (A = Rb Cs; X = F, Cl, Br, I) have been widely investigated in the literature in recent years [37,38,39,40,41,42,43]. Additionally, the crystal structure and electrical transport properties of AZn_2_Sb_2_ (A = Ca, Yb) have been studied experimentally in 2012 [44]. Recently, an investigation on the optoelectronic characteristics and elastic properties of YbZn_2_X_2_ (X = N, P, As, Sb) was conducted by our group [45]. This gives us an idea that the replacement of the alkaline earth metal cations by Yb^2+^ is possible as another choice for chalcogenide perovskites. To our knowledge, no experimental and theoretical studies have reported the properties of Yb-containing chalcogenide perovskites. Therefore, this also prompts us to explore the phase stability and optoelectronic properties of this new class of Yb-containing chalcogenides.

In this study, we have theoretically investigated the stability, mechanical behavior, optoelectronic properties, electron transport, and photovoltaic performance of YbMX_3_ (M = Zr, Hf; X = S, Se) via first-principle calculations. Through our detailed and comprehensive analysis, it is revealed that all compounds are mechanically and dynamically stable, and they are ductile materials. The results further disclose that these excellent properties—including the suitable band gap, high electron transport, and strong visible-light absorption coefficient—are revealed for YbZrSe_3_ and YbHfSe_3_. The analysis of the simulated maximum conversion efficiency reveals that YbMSe_3_ (M = Zr, Hf) is an excellent candidate for single-junction solar cells. More importantly, YbZrSe_3_ exhibits the highest efficiency of ~32%. Our study can provide theoretical guidance for further experimental exploration of the optoelectronic properties and photovoltaic performance of Yb-based chalcogenide perovskites.

## 2. Computational Details

All calculations were carried out based on the density functional theory (DFT) as implemented in the Vienna ab initio simulation package (VASP 5.4.4) [46]. The projector augmented wave (PAW) [47] method was used to describe the ion–electron interactions. The Perdew–Burke–Ernzerhof (PBE) functional of the generalized gradient approximation (GGA) was applied to treat the exchange–correlation potential [48]. The convergence criteria of the force on each atom and energy were lower than 10^−2^ eV/Å and 10^−6^ eV, respectively. A-centered 7 × 6 × 7 *k*-point grid and 600 eV cutoff energy were employed for calculating the material properties. The phonon spectra were simulated at 0 K by using the finite displacement method based on the PHONOPY package 2.17 [49]. The electronic structures and optical properties were precisely obtained by the Heyd–Scuseria–Ernzerhof (HSE06) hybrid functional [50]. The photovoltaic performance was quantified by employing the spectroscopic limited maximum efficiency (SLME) method [51], and the detailed computations were described in the recent literature [30,31]. The crystal structure was visualized by VESTA 3.5 [52], and all the data were further gained by using VASPKIT 1.5 [53].

## 3. Results and Discussion

### 3.1. Structural Characteristics

**Figure 1:**
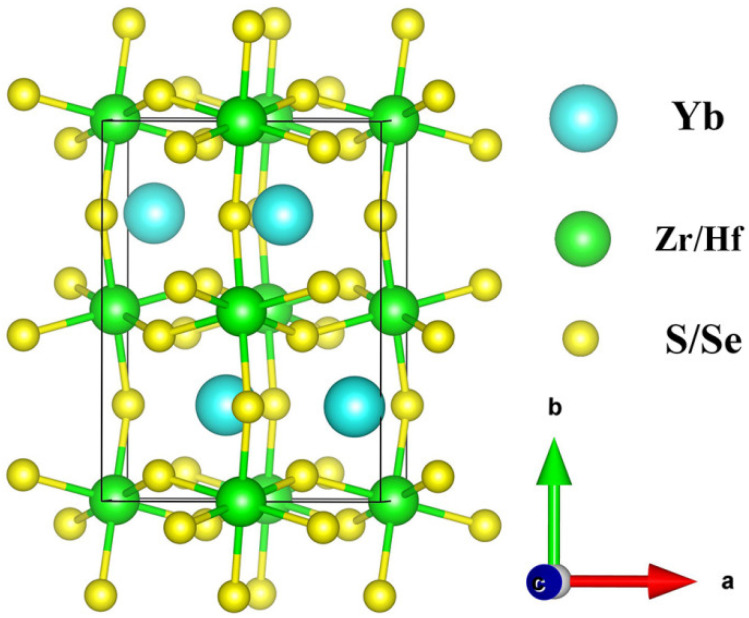
Orthorhombic crystal structure of YbMX_3_ (M = Zr, Hf; X = S, Se).

The three-dimensional orthorhombic perovskite structure with the *Pnma* space group is constructed herein for YbMX_3_ (M = Zr, Hf; X = S, Se) by substituting Ca atoms with Yb atoms in the case of Ca(Zr/Hf)(S/Se)_3_ because of the similar ionic radii of Yb^2+^ and Ca^2+^ [54]. The orthorhombic crystal structure of YbMX_3_ (M = Zr, Hf; X = S, Se) is demonstrated in Figure 1, where the Yb cation is twelve coordination and the Zr or Hf cation is surrounded by six anions [33]. The obtained lattice parameters of four compounds are illustrated in Table 1. The lattice parameters of YbZrS_3_ and YbZrSe_3_ are very close to those of Ca-based analogues [35]. The lattice constant and volume are significantly increased from YbMS_3_ to YbMSe_3_ due to an increase in the ionic radius from S^2−^ (1.84 Å) to Se^2−^ (1.98 Å). Additionally, it is worth noting that the lattice parameters of YbHfX_3_ are slightly smaller than that of YbZrX_3_ owing to the reduction of the ionic radius from Hf^4+^ (0.71 Å) to Zr^4+^ (0.72 Å).

### 3.2. Stability Evaluation

**Figure 2:**
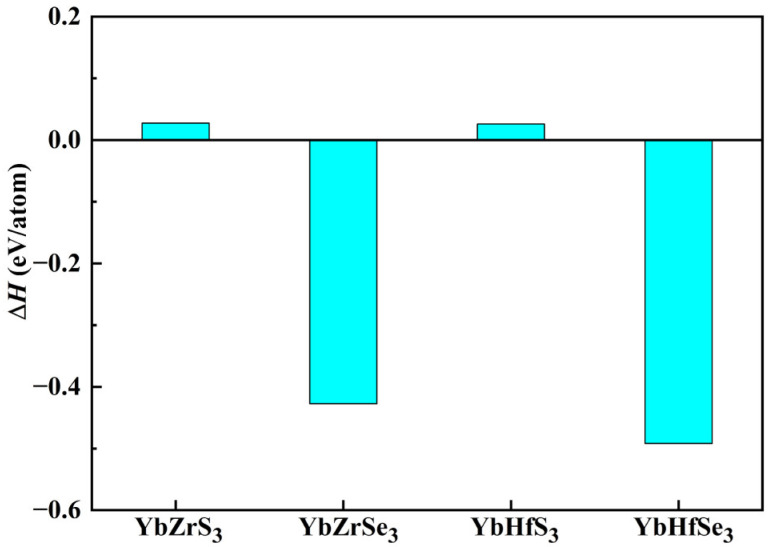
The calculated formation energy of YbMX_3_ (M = Zr, Hf; X = S, Se).

**Figure 3:**
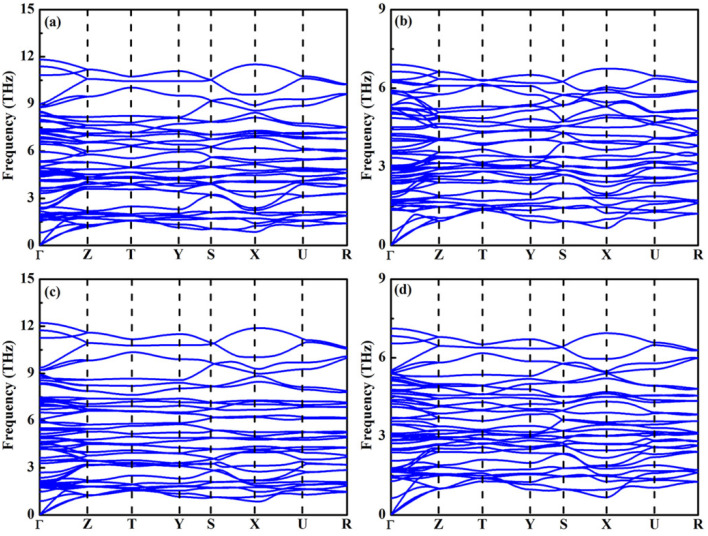
The phonon dispersion spectra of (**a**) YbZrS_3_, (**b**) YbZrSe_3_, (**c**) YbHfS_3_, and (**d**) YbHfSe_3_.

It is well known that only when the new material shows good structural stability in theory can it be proven that it has the conditions to be synthesized experimentally. Therefore, the stability of four novel chalcogenide perovskites YbZrS_3_, YbZrSe_3_, YbHfS_3_, and YbHfSe_3_ were evaluated by its thermodynamic stability, dynamic stability, and mechanical stability. To study the thermodynamic stability of four novel materials, the formation energy (Δ*H*) calculations are conducted by the following decomposition path:

(1) $$\Delta H_{1}=E({\mathrm{YbMX}}_{3})-E(\mathrm{YbX})-E({\mathrm{MX}}_{2})$$

where *E*(YbX) and *E*(MX_2_) are the total energies of bulk YbX and MX_2_ (M = Zr, Hf; X = S, Se) as the same calculations are performed. The lowest energy experimental crystal structure (such as YbS, YbSe, ZrS_2_, ZrSe_2_, HfS_2_, and HfSe_2_) was adopted from the Materials Project database [55]. In Figure 2, the results illustrate that both YbZrS_3_ and YbHfS_3_ are marginally stable while YbZrSe_3_ and YbHfSe_3_ are highly stable. It is noted that YbZrS_3_ and YbHfS_3_ are still synthesized under the appropriate experimental conditions, which is similar to the case of CaZrS_3_ [35]. In the case of YbMX_3_ (M = Zr, Hf), the stability is significantly enhanced by the substitution of S with Se. The phonon spectra of four unreported materials under ambient pressure are depicted in Figure 3. It is clear that there is no imaginary frequency in the whole Brillouin zone, implying that they are dynamically stable.

The crystal structure is considered to be mechanically stable when their elastic constants meet with the Born stability criteria. There are nine independent elastic constants, which is consistent with the characteristics of an orthorhombic crystal structure, so the following Born stability conditions need to be satisfied [56]:

(2) $$\begin{array}{l} C_{11}>0,C_{11}C_{22}>C_{12}^{2},C_{44}>0,C_{55}>0,C_{66}>0\\ C_{11}C_{22}C_{33}+2C_{12}C_{13}C_{23}-C_{11}C_{23}^{2}-C_{22}C_{13}^{2}-C_{33}C_{12}^{2}>0 \end{array}$$

Table 2 displays the elastic constants of four new compounds. The mechanical stability criteria are all satisfied, indicating that YbZrS_3_, YbZrSe_3_, YbHfS_3_, and YbHfSe_3_ are mechanically stable at ambient pressure. The important elastic moduli (bulk modulus *B*, shear modulus *G*, and Young’s modulus *Y*) are further obtained by the Voigt–Reuss–Hill (VRH) approximation [57]. These elastic moduli are also listed in Table 2. The elastic moduli *B*, *G*, and *Y* represent the compressibility, the shear deformation, and the stiffness of solid materials, respectively. The three elastic moduli of YbHfS_3_ are all the largest, indicating that this compound has the strongest capacity to oppose compression and shear deformation, and it is the stiffest material. On the contrary, YbZrSe_3_ has the three smallest elastic moduli, and it shows better flexibility. The ductility and brittleness of these chalcogenide perovskites were further explored by calculating their Pugh’s ratio (*B/G*) and Poisson’s ratio (*v*) [58]. The critical values are 1.75 for Pugh’s ratio and 0.26 for Poisson’s ratio, respectively. The obtained values of Pugh’s ratio and Poisson’s ratio of YbZrS_3_, YbZrSe_3_, and YbHfS_3_ are higher than the above critical values, implying that they are ductile materials. On the contrary, YbHfSe_3_ is a brittle material.

### 3.3. Electronic Properties

**Figure 4:**
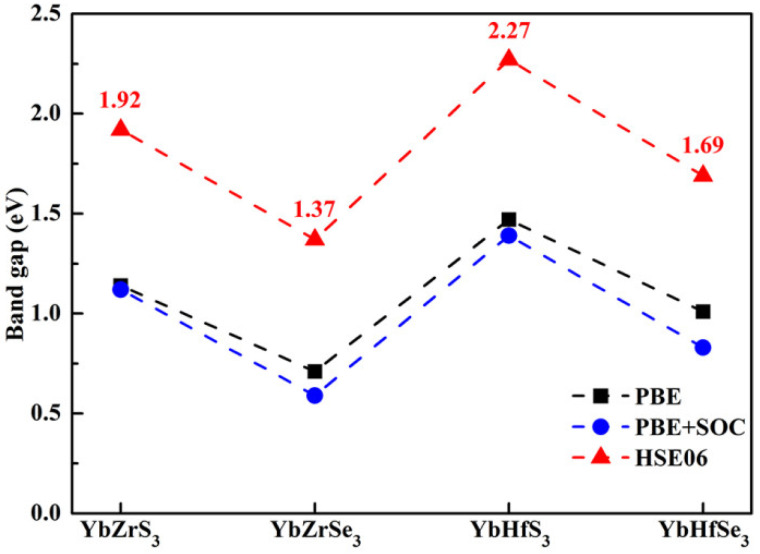
The band gap variation of YbMX_3_ (M = Zr, Hf; X = S, Se) with different methods.

**Figure 5:**
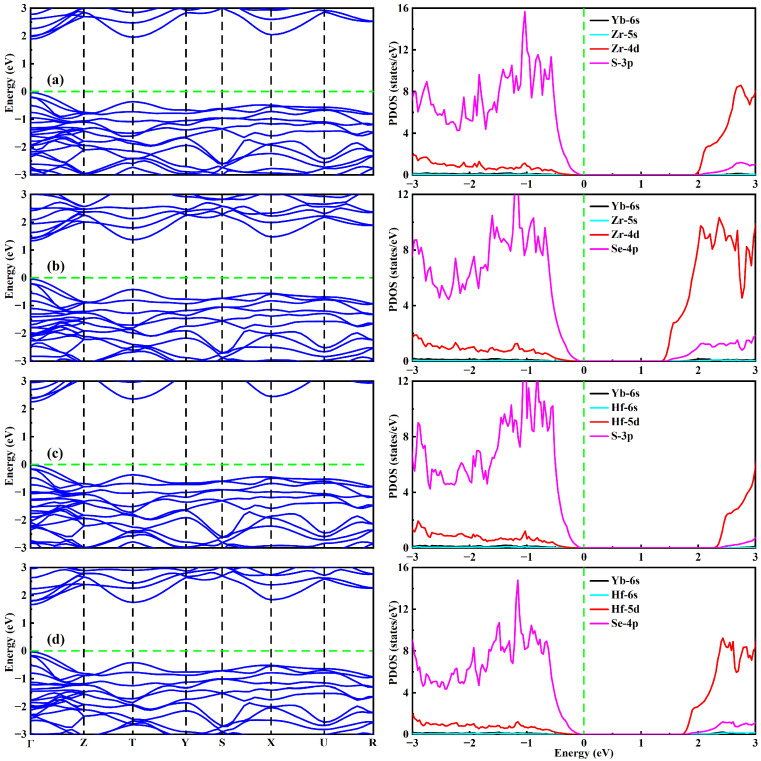
Band structures and partial density of states of (**a**) YbZrS_3_, (**b**) YbZrSe_3_, (**c**) YbHfS_3_, and (**d**) YbHfSe_3_. The green dashed line corresponds to the Fermi energy level.

According to the Shockley–Queisser (SQ) limit theory [59], the electronic band gap of a material is a crucial parameter for its photovoltaic application. The theoretical maximum efficiency of a material with 1.34 eV is ~33% for a single-junction solar cell [59]. To further study the electronic properties of YbMX_3_ (M = Zr, Hf; X = S, Se), their electronic band structures were calculated along with high symmetry points in the Brillouin zone by using the PBE and HSE06 functionals. It is observed from Figure 4 that the similar energy gap variation curves are illustrated for two different methods. It was verified from our recent theoretical report [35] that the optical energy gaps calculated by the PBE functional of Zr-based chalcogenide perovskites are significantly lower than the experimental data. Additionally, the spin–orbit coupling (SOC) effect is further explored herein. After considering the SOC effect, the band gap reduction values are 20 meV for YbZrS_3_, 120 meV for YbZrSe_3_, 80 meV for YbHfS_3_, and 180 meV for YbHfSe_3_. The results indicate that the SOC effect is relatively small (<200 meV) for YbMX_3_ (M = Zr, Hf; X = S, Se). It is mentioned that the band gap energy of AZrS_3_ (A = Ca, Sr, Ba) can be reproduced well by employing the HSE06 hybrid functional in comparison with the experimental observation data [21,35]. Moreover, owing to the small SOC effect and the considerably time-consuming HSE06-SOC calculation, the SOC effect is not included in this study. It is clearly seen from Figure 5 that four compounds display direct-gap characteristics at the high symmetry point Γ. The direct-gap values are 1.92 eV for YbZrS_3_, 1.37 eV for YbZrSe_3_, 2.27 eV for YbHfS_3_, and 1.69 eV for YbHfSe_3_. The band gap reduction can reach 0.55 eV and 0.58 eV for the cases of YbZrX_3_ and YbHfX_3_, respectively, via the substitution of S with Se. Our simulation results indicate that YbZrSe_3_ is predicted to be an excellent candidate for high-efficiency single-junction solar cells. Furthermore, the band gap feature of YbZrX_3_ is analogous to the case of AZrX_3_ (A = Ca, Sr, Ba) [35].

The partial density of states (PDOS) of four compounds are demonstrated in Figure 5. The detailed PDOS analysis can provide crucial information on the orbital contributions of various atoms. It can be seen that the valence band (VB) edge of YbMX_3_ is mostly composed of the 3p/4p orbitals of the S/Se atoms, while the conduction band (CB) edge is mainly composed of the 4d/5d orbitals of the Zr/Hf atoms. The Yb–6s, Zr–5s, and Hf-6s orbitals have little contribution to the VB and CB edges. Therefore, the electronic structure of YbMX_3_ is mainly determined by the chemical bonds M–X originating from the hybridization between the Zr–4d/Hf–5d and S–3p/Se–4p orbitals.

### 3.4. Carrier Transport Ability

The effective masses of electrons and holes are significant for the photovoltaic performance of a material. We have calculated the carrier effective masses of four compounds along with the Γ→Z direction based on their band structures by using the formula $m*=\hbar^{2}{\left[\frac{\partial^{2}E(k)}{\partial k^{2}}\right]}^{-1}$ [60], where *ħ* is the reduced Plank constant, *E*(*k*) is the eigenvalue of the energy band, and *k* is the wave vector along different directions. In addition, the exciton binding energy is another important factor for the electron transport, and this value is computed by the Mott–Wannier model given by the equation $E_{b}=\frac{13.56\mu }{\varepsilon_{0}^{2}}$($\mu =\frac{m_{e}^{*}\times m_{h}^{*}}{m_{e}^{*}+m_{h}^{*}}$) [61], where *μ* and *ε*_0_ are the reduced effective mass and static dielectric constant, respectively. The calculated values are listed in Table 3. It has been found that all compounds have relatively large electron and hole effective masses with 0.52–0.58 *m*_0_ and 0.55–0.70 *m*_0_ in comparison with those of Pb-based halide perovskites [62,63]. The exciton binding energy is dramatically decreased from YbMS_3_ to YbMSe_3_. The above analysis shows that YbZrSe_3_ has the lowest effective masses and exciton binding energy, which is valuable for the carrier transport. Therefore, YbZrSe_3_ is more suitable as an ideal light-absorbing material.

### 3.5. Optical and Photovoltaic Performance

**Figure 6:**
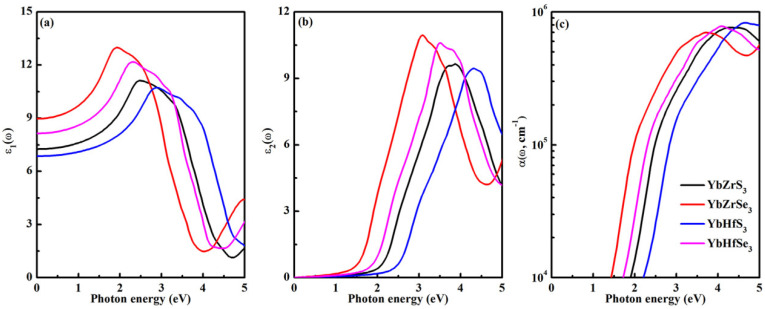
The calculated optical properties of YbMX_3_ (M = Zr, Hf; X = S, Se): (**a**) *ε*_1_(ω), (**b**) *ε*_2_(ω), and (**c**) *α*(ω).

The excellent optical properties of materials are significant for achieving a high efficiency of photovoltaic application. The optical characteristics of four novel compounds are further studied to reveal their potential for single-junction solar cells. We have calculated the real (*ε*_1_) and imaginary (*ε*_2_) parts of the frequency-dependent dielectric function *ε*(ω), as shown in Figure 6a,b. The static dielectric constant *ε*_1_(0) varies notably with chemical composition, which is inversely proportional to the band gap of the material. The high dielectric constant is beneficial for the carrier transport, such as YbZrSe_3_ and YbHfSe_3_. It can be deduced from Figure 6b that the optical absorption curves of four compounds are different from each other. YbMX_3_ has an absorption edge that is very close to its direct band gap, suggesting that there is an efficient direct optical transition from the VB to CB. Figure 6c shows that the absorption coefficient increases dramatically as the photon energy approaches the fundamental direct gap. From YbMS_3_ to YbMSe_3_, the significant improvement of light absorption capacity is realized. The remarkable light absorption attenuation feature is observed from YbZrX_3_ to YbHfX_3_. These optical features are close to their electronic band structures. Markedly, YbZrSe_3_ and YbHfSe_3_ exhibit high visible-light absorption coefficients, implying their great potential applications for optoelectronic devices.

**Figure 7:**
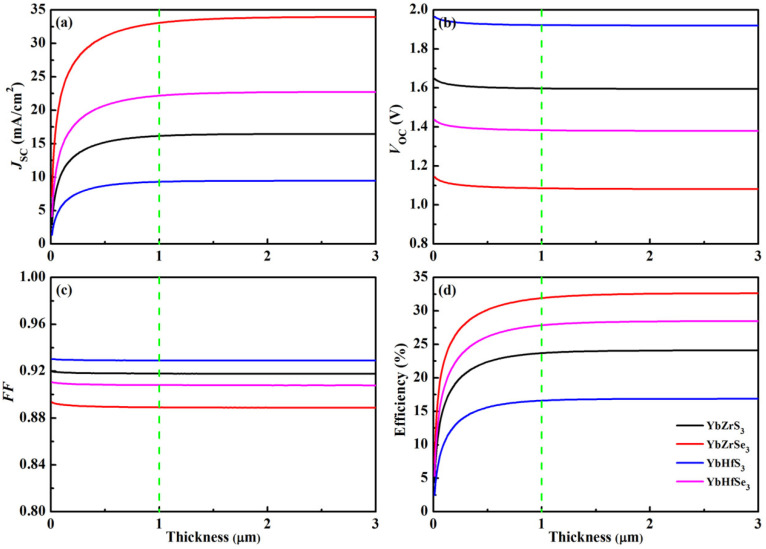
The various photovoltaic parameters of YbMX_3_ (M = Zr, Hf; X = S, Se) as a function of the absorber layer thickness: (**a**) *J*_SC_, (**b**) *V*_OC_, (**c**) *FF*, and (**d**) efficiency.

The optimal direct-gap and high visible-light optical absorption of YbZrSe_3_ indicate its high efficiency. As an improvement of the SQ model, the advanced SLME method was developed by Yu and Zunger [51] to evaluate the photovoltaic performance in theory. In the SQ model, the promising candidate is only chosen on the basis of its band gap. However, the SLME method overcomes the SQ model’s shortcoming by taking into consideration the band gap with its nature, the optical absorption, the recombination mechanism, and the film thickness. The SLME method has been successfully used to predict the conversion efficiencies of different types of chalcogenide perovskites in recent years [30,31]. The curves of the short-circuit current density *J*_SC_, the open-circuit voltage *V*_OC_, the fill factor *FF*, and the efficiency of four compounds as a function of film thickness are plotted Figure 7. The photovoltaic parameters remain almost unchanged and reach their maximum values when the absorption layer thickness exceeds 1 μm. Table 4 summarizes the photovoltaic parameters of four compounds with the film thickness of 1 μm. It can be seen that the efficiency of a material is closely related to its band gap and absorption coefficient. The maximum conversion efficiency of YbHfS_3_ is less than 17% due to its smallest *J*_SC_ value (9.26 mA/cm^2^). Three compounds YbZrS_3_, YbZrSe_3_, and YbHfSe_3_ have maximum efficiencies beyond 23%. It is mentioned that the efficiency can reach up to ~28% for YbHfSe_3_. More importantly, YbZrSe_3_ shows the highest efficiency of 31.90% with *J*_SC_ = 33.06 mA/cm^2^, *V*_OC_ = 1.09 V, and *FF* = 88.9%. Additionally, the maximum efficiency of YbZrSe_3_ is comparable to those (~31%) of halide perovskite CH_3_NH_3_PbI_3_ [64] and the inorganic materials GaAs and CdTe [65]. All these results suggest that YbZrSe_3_ is an excellent promising candidate for photovoltaic materials in single-junction solar cells.

## 4. Conclusions

In summary, we have conducted a detailed, comprehensive investigation of the stability, mechanical, electronic, optical, and photovoltaic properties of lead-free novel chalcogenide perovskites YbMX_3_ (M = Zr, Hf; X = S, Se) by using first-principle calculations. It is observed that YbZrSe_3_ and YbHfSe_3_ exhibit superior phase stability against decomposition while YbZrS_3_ and YbHfS_3_ are marginally thermodynamically stable. Moreover, the dynamical and mechanical stability of Yb-based chalcogenide perovskites are further verified, and they are all ductile materials. The effects of quaternary metal cations and anions on the electronic and optical properties of chalcogenide perovskites are elucidated in detail. The substitution of S with Se is discovered to have the ability to reduce the band gap by about 0.6 eV. The band gap is increased from YbZrX_3_ to YbHfX_3_ because the 4d orbitals of Zr are lower than the 5d orbitals of Hf. The HSE06-based electronic structure calculations show that YbZrSe_3_ and YbHfSe_3_ possess direct band gaps with variation from 1.37 to 1.69 eV, indicating that they show great potential for photovoltaic devices. The excellent electron transport ability is elucidated for YbZrSe_3_ and YbHfSe_3_. The optical and photovoltaic properties of these compounds are further revealed. The results show that YbMSe_3_ (M = Zr, Hf) exhibits high conversion efficiency beyond 27%, especially for YbZrSe_3_, whose efficiency can reach ~32%. Our theoretical discovery can inspire further experimental research exploring the photovoltaic performance of novel Yb-based chalcogenide perovskites.

## Figures and Tables

**Table 1:** The optimized lattice parameters for YbMX_3_ (M = Zr, Hf; X = S, Se).

Compound	*a*/Å	*b*/Å	*c*/Å	*V*/Å^3^
YbZrS3	7.07	9.60	6.52	442.15
YbZrSe3	7.39	10.04	6.79	504.31
YbHfS_3_	6.70	9.52	6.49	432.45
YbHfSe_3_	7.33	9.98	6.77	495.21

**Table 2:** The various elastic properties of YbMX_3_ (M = Zr, Hf; X = S, Se).

Parameter	YbZrS_3_	YbZrSe_3_	YbHfS_3_	YbHfSe_3_
*C*_11_ (GPa)	154.5	127.3	163.9	134.7
*C*_12_ (GPa)	38.4	30.5	39.4	31.2
*C*_13_ (GPa)	55.7	44.1	58.9	46.5
*C*_22_ (GPa)	135.3	113.5	148.9	123.5
*C*_23_ (GPa)	35.3	27.3	35.8	27.5
*C*_33_ (GPa)	97.6	77.9	105.3	84.2
*C*_44_ (GPa)	23.0	18.2	26.6	21.5
*C*_55_ (GPa)	43.4	36.9	49.7	42.0
*C*_66_ (GPa)	43.3	36.0	47.3	39.0
*B* (GPa)	70.4	56.7	74.8	60.1
*G* (GPa)	37.4	31.0	41.8	34.6
*Y* (GPa)	95.3	78.6	105.8	87.2
*B/G*	1.882	1.832	1.789	1.735
*ν*	0.274	0.269	0.264	0.258

**Table 3:** Computed effective masses of electron and hole, reduced effective mass, static dielectric constant, and exciton binding energy of YbMX_3_ (M = Zr, Hf; X = S, Se).

Absorber	*m*_e_* (*m*_0_)	*m*_h_* (*m*_0_)	*μ* (*m*_0_)	*ε*_0_	*E*_b_ (meV)
YbZrS_3_	0.578	0.678	0.312	7.254	80
YbZrSe_3_	0.542	0.553	0.274	8.972	46
YbHfS_3_	0.549	0.705	0.309	6.854	89
YbHfSe_3_	0.520	0.597	0.278	8.138	55

**Table 4:** The simulated photovoltaic parameters of YbMX_3_ (M = Zr, Hf; X = S, Se).

Absorber	*J*_SC_ (mA/cm^2^)	*V*_OC_ (V)	*FF* (%)	Efficiency (%)
YbZrS_3_	16.15	1.60	91.8	23.68
YbZrSe_3_	33.06	1.08	88.9	31.90
YbHfS_3_	9.26	1.92	93.3	16.59
YbHfSe_3_	22.17	1.38	91.0	27.84

## Data Availability

The original contributions presented in this study are included in the article. Further inquiries can be directed to the corresponding authors.
